# The American Dental Society of Europe

**Published:** 1890-08

**Authors:** 


### THE AMERICAN DENTAL SOCIETY OF EUROPE.—SEVENTEENTH ANNUAL MEETING, PARIS, AUGUST 6 AND 7, 1889.

(Continued from page 245.)

*Dr. Mitchell.*—Mr. President, through some mistake Dr. Haskell's paper was left behind, and as I know the important features of it, I will do my best to present them to you. Dr. Haskell's paper was principally historical in connection with continuous-gum work. He spoke of Dr. Allen's first introduction of continuous-gum work in Boston, a little over thirty years ago. Dr. Allen was very enthusiastic in regard to this work, according to the light of the profession at that time, and I may say it has extended down quite to the present period. Dr. Allen's work originally consisted, not of making the continuous gum as we have it now, but of soldering on the teeth, not quite so effectually as at present, and sometimes the body was carried around the pins of the teeth and down over the footings of the backing to make it comfortable to the tongue; but beyond that there was no attempt to make the plate and produce the rugæ as now practised. There were a great many dentists in Boston who adopted this with the usual degree of enthusiasm incidental to new converts. As there was much to be learned, they soon found it was not quite so easily manipulated as they had supposed. Dr. Haskell made several changes in the character of the work, and he assisted very materially in improving the material of the gum body. He found that this process uncombined was a very good thing, but does not believe in it when connected with vulcanite. Dr. Haskell also introduced what he terms a "doubler." I was not aware of his use of this. I adopted the same method and found it an exceedingly good thing. This is produced by waxing up to the required thickness, making model and dies as required, and striking up a semicircular piece of platinum and soldering it to the back end of the plate and flowing the body underneath that. It is well known that in continuous gum we have a work which is only limited in its artistic capacity by that of the operator, and it is one that you cannot delegate to another; every one should do the labor himself. Another point should be mentioned here, and which has been emphasized by Dr. Haskell again and again, and that is, do not be afraid to carry a plate too high where the cuspids have been extracted to secure proper muscular action at these points and relieve the depression, which is always present at the side of the nose in persons wearing an artificial case. The appreciative capacity for anatomical harmony is lacking in the ordinary workman, and the dentist cannot relegate this work to another.

A great many objections have been raised in regard to the weight of the work; but weight in a lower plate is a great advantage, and, in the majority of cases, it is not a serious matter if the plate fits. As soon as the plate breaks loose from atmospheric pressure the weight is felt. If there is a perfect adaptation, a thorough knowledge of the requirements of the case, and a modification of the model as the occasion may require, a perfect sense of comfort is insured to the patient. The general plan of masticating surfaces can certainly be secured in a manner superior, or equal, to that of any other method, from the fact that plain or single teeth are used.

You can depress a tooth, elevate, lengthen, or shorten; in fact, it is capable of any modification. In regard to the manipulation in detail, this is familiar to all of you, and it is therefore unnecessary to describe it at length.

There is one thing more I wish to mention. As there seems to be some hesitation in selecting the best means of baking plates, a certain number of furnaces have been introduced. Dr. Verrier's I have used, and I must say it has not been very successful; indeed, he himself has not met with the success he could have wished. The heat must be brought gradually, steadily, but surely directly to the fusing point, and to be allowed to remain there so as to permit the particles to coalesce thoroughly, for just in proportion as you get a rapid fluctuation of heat is unequal expansion and contraction promoted, producing crazing, which is one of the most marked imperfections of the work. I would strongly recommend a coke furnace. Dr. Haskell has adapted the coke furnace to the requirements of the ordinary laboratory by surrounding it with asbestos cloth, to prevent radiation.

In long articulations, where the teeth are far removed from the maxillary ridge, you get a leverage, and in prognathous cases there is a tendency of the teeth to fall out of position during baking. This is more liable to occur where the heat rises and falls rapidly, so you will see that the great desideratum is to have a furnace where the heat can be controlled perfectly. Dr. Land, of Detroit, Mich., U. S., has produced a gas furnace for which much is claimed, but of its practical workings I have had no experience. I feel quite sure that continuous gum work is a branch of prosthetic dentistry that every one with a little patience and a little practice can manipulate successfully.

The following reply to the telegram sent to Saratoga, United States, was received:

"PATTON, SECRETARY A. D. S. OF EUROPE, PARIS.

"The American Dental Association reciprocate kindly greetings of the American Dental Society of Europe.

"CUSHING, *Secretary.*"

### *Second Day.—Afternoon Session.*

Dr. Miller's histological studies by aid of the lantern.

### DR. ELLIOTT ON ANTIFEBRIN.

GENTLEMEN,—At the time I took up this subject, six months ago, it was to me quite new; my attention was called to it by a physician who was using it very largely in neuralgia. In my opinion we have no remedy which approaches in value this agent. I have used it in fifty cases, and in forty-five of them have been successful, —that is, in reducing within a few hours the pain of advanced periodontitis.

I cannot give you accurate details, because I could not carry the record as far as I wished, but, in my experience, it is superior to the local application of either aconite or iodine.

I always confine my treatment to two doses of ten grains each, and the pain is almost immediately relieved, and there are no ill effects from it as from the use of opiates. I took up the use of another substance, phenacetin, a medicine of light character, but the antifebrin has been the most successful.

I would like to call your attention to some experiments I have made with amalgams. The main object of these *experiments* was to determine what part each metal took, which was the expanding and which was the contracting metal, and what proportion of these metals was necessary to get the best results. It has been stated in Dr. Flagg's book that silver was the expanding agent, while tin contracted; his views have, in a measure, been confirmed by Dr. Kirby, of Bedford, England. Dr. Flagg, as I remember, states it merely as a fact and does not give us his proofs, while Dr. Kirby seems to have demonstrated it by frequently measuring with a micrometer a bar of silver amalgam and also by the breaking of glass tubes; but, of course, tests of this kind cannot be considered to be very accurate from their very nature. I have made a number of experiments by the specific gravity test, which, as you know, is extremely delicate, so much so that I have found it quite unnecessary to consider the question of equable heat. In my earlier tests I kept the amalgam under investigation in water, heated, and kept at a temperature of 100° F., but I found that in a mass, say of ten grains, the difference in bulk between 60° and 100° was only some three milligrammes, or as 3 to 10,000. Consequently I made no further effort to keep the water warm.

Now, in regard to silver. I took masses of from five to ten grammes, weighed them in water, sometimes every few hours, at other times only once or twice a day, but the result was always the same contraction in nearly every case; now, whether the copper had anything to do with this or not I do not know. I used sterling silver, which contains eight per cent. of copper. You will see by the table the amount of contraction.

TABLE.

*Standard Silver Amalgam.*

| 1889. June | Standard Silver. | | |
|---|---|---|---|
| | Medium grain. | Coarse grain. | Fine grain. |
| 18 | 5.966 | 6.598 | 5.387½ |
| 19 | 5.989 | 6.616 | 5.361½ |
| 20 | 5.998 | 6.619 | 5.385 |
| 21 | 5.996 | 6.622 | 5.385½ |
| 22 | 5.998 | 6.624 | 5.385 |
| 24 | 5.999½ | 6.629 | 5.386 |

Now, in regard to the combination of silver with other metals, I have experimented with tin, zinc, bismuth, aluminium, etc. The tables of the first three are before you, as per example.

*Silver and Tin.*

| 1889. June | No. 1. Standard Silver and 5 per cent. tin. | No. 2. Standard Silver and 10 per cent. tin. | No. 3. Standard Silver and 15 per cent. tin. | No. 4. Standard Silver and 20 per cent. tin. |
|---|---|---|---|---|
| 13 | 4.978 | 4.420 | 3.288 | 2.980 |
| 14 | 5.044 | 4.442 | 3.279½ | 3.036 |
| 15 | 5.053 | 4.451 | 3.418 | 3.055 |
| 17 | 5.082 | 4.465 | 3.456 | 3.080 |
| 18 | 5.091 | 4.470½ | 3.466 | 3.095 |
| 19 | 5.098½ | 4.470 | 3.469½ | 3.096½ |

*Silver and Zinc.*

| 1889. June | No. 1. Standard Silver and 5 per cent. zinc. | No. 2. Standard Silver and 10 per cent. zinc. | No. 3. Standard Silver and 15 per cent. zinc. | No. 4. Standard Silver and 20 per cent. zinc. |
|---|---|---|---|---|
| 14 | 6.722 | 5.410 | 4.181 | Too friable to manipulate. |
| 15 | 6.748 | 5.473 | 4.210 | |
| 17 | 6.762 | 5.469 | 4.210 | |
| 18 | 6.764 | 5.475 | 4.206 | |
| 19 | 6.764 | 5.477 | 4.208 | |
| 20 | 6.767 | 5.477 | 4.210 | |

*Silver and Bismuth.*

| 1889. June | No. 1. Standard Silver and 5 per cent. bismuth. | No. 2. Standard Silver and 10 per cent. bismuth. | No. 3. Standard Silver and 15 per cent. bismuth. | No. 4. Standard Silver and 20 per cent. bismuth. |
|---|---|---|---|---|
| 11 | 4.496 | | | |
| 12 | 4.560 | 5.830 | 5.582 | |
| 13 | 4.570½ | 5.880½ | 5.706½ | 5.550 |
| 14 | 4.579 | 5.896½ | 5.748 | 5.638 |
| 15 | 4.588 | 5.909 | 5.748½ | 5.671 |
| 17 | 4.601½ | 5.927 | 5.774 | 5.642 |

My experiments with aluminium are quite negative. When combined with silver its power of amalgamation is infused in direct proportion to its amount, and can only be used, practically, when it has less than five per cent. of the alloy. No good property has been developed to encourage further investigation. It does not follow that, because two metal alloys contract, the addition of a third contracting metal will increase that contraction. We cannot tell by any process of reasoning what the result will be. In conclusion, let me say that while my experiments with amalgam may not be directly useful, they give us some little insight into the complex problem before us.

The great difficulty in making these experiments—or rather of getting some practical good from them—is the uncertain nature of the materials made use of. We are never sure of getting the same alloy twice, owing to the almost impossibility of getting pure metals and the effect on the material by different modes of treatment. I have not found that the physical condition of the alloy was important in relation to the question of contraction. Experiments with coarse, medium, and fine grains did not develop any facts of interest; while the fine was pleasanter to work and would probably prove the most useful in practice, the coarse was not inferior.

Allow me to show you a new reducing and regulating stop-valve I have designed and made for use with the oxyhydrogen blow-pipe and lantern work; also a sand-paper disk-holder, new instruments, and appliances; hand-piece, right-angle; mechanical mallet, straight and right-angle; special burs, amalgam instruments, etc. I take this opportunity of exhibiting them, for, having retired from practice, it is not likely that I will again have the pleasure of meeting you.

Those who were present at our last meeting will remember that Dr. Young brought forward some gutta-percha impressions, and they were certainly far superior to anything I have ever seen. I have failed to get the same results with the same material. Dr. Young even was not successful in my own mouth. We failed to get the material in England, and Dr. Jenkins was good enough to send it to me from Germany. It has one objection, however, although it is my favorite material, and that is the length of time it takes to harden. I tried some experiments to get at certain facts, for we are too easily led to follow the experience of others instead of trying for ourselves. In regard to Stent's and other preparations, there is a temperature at which they work best; if you go higher you do not get good results; if you use red gutta-percha, it is too low.

I also have some specimens of gutta-percha stoppings which have no particular value, but simply are shown as an evidence of how easy it is for you to make your own gutta-percha; it costs about two dollars a pound instead of five dollars an ounce; it is made, in spite of Dr. Flagg's elaborate method, of gutta-percha and oxide of zinc, and you roll in from three to six per cent.

I also brought a pivot with which I have had ten years' experience, and it has proved itself good. I use a wedge pin to secure it; this specimen which I have here will explain its advantages in certain cases.

*Dr. Mitchell.*—In regard to your amalgam test, have you considered barometric pressure as having any bearing upon such delicate test?

*Dr. Elliott.*—You may not know that the test is an exceedingly delicate one; if you heat a tooth ten degrees, Fahrenheit, of course, it would be quite different. Actual changes of temperature can be quite easily discarded from your calculations. Some say, Why don't you make the test in water, which can be kept at any temperature.

I have done so, and it made a difference of about three milligrammes.

*Dr. Mitchell.*—Speaking of the antifebrin, I remember you telling me of its benefits. I undertook a series of experiments, and I can thoroughly endorse its good qualities. I have a remedy, but I do not think it gives the same results as the antifebrin, and that is quinine. I use it a great deal in my practice here, in doses from four to ten grains, given at night; two grains at intervals of an hour, until the desired dose is administered; if there is any slight disturbance, quinism being produced, I find that the action of quinine is, if anything, more accurate than the antifebrin. There are many persons who can take the antifebrin who cannot take quinine.

*Dr. Elliott.*—I hold a piece of metal manufactured by a patient of mine, really an improved brass, made of copper and zinc with a very small proportion of iron. I have made a plate, which has been worn by my secretary for some time, and it answers very well as far as I have observed. It is exceedingly thin, but it has held its position as a suction plate. Of course, it is only intended for very common and cheap work; the copper being cheap, it can be soldered with silver solder.

*Dr. Kelsey.*—Can any one tell us what is the character of the new metal which resembles platina? Is it not composed of nickel?

*Dr. Elliott.*—Dental alloy, I think it is, and it is composed of silver and platinum.

*Dr. Mitchell.*—Would not the nickel, which has a stiffness about it not possessed by any alloy, prove a great disadvantage to its working qualities? Dental alloy is a combination of silver and platinum. Before reducing it quite to the required thickness, the silver is eaten off the surface by acid, and the platinum in the subsequent rolling is diffused entirely over the surface. I think the combination of silver and nickel would make a very safe preparation for working, owing to its less liability to tarnish and the sulphides forming less readily upon it.

*Dr. Bonwill.*—If any one takes the INTERNATIONAL DENTAL JOURNAL, you will see in the January and February numbers the method of retracting, and at the same time keeping teeth in position. It is a double band, which you can see is fastened round the teeth, working one inside and out of the other. The pressure is produced simply by a piece of rubber tubing, thus enlarging the arch and at the same time drawing those teeth in or out of position.

Now, I have another little device, which is used to reduce to any infinitesimal size nerve broaches, which we all find so necessary.

It is nothing more than taking a soft steel or rubber packing and placing between them emery disks, and by placing the small broach in between this and revolving it, it cuts it down to any size desired. If you try it, you will find it one of the most useful things you ever had in your office, and the means of removing the pulp from the finest canals. It has given me more satisfaction than anything else I have in my office.

*Dr. Du Bouchet.*—I have not prepared any paper on the following subject, but the other day I came across these models, and I thought it would be of some interest to the Society. I regret I cannot show you the living subject. A man, fifty years of age, came to me; the jaws were flattened in the middle, and there was a contraction which increased more and more every day, and when he came to me the mouth was almost closed. I could not take an impression in that condition, and in order to expand the jaw it was necessary to have an impression. I had him come a day or two later, and instructed him how to use his fingers to enlarge somewhat the space. He tried this for a week, and all the tissues yielded. I was then able to introduce half an impression-cup in the mouth, but it was not the right size. I took a piece of wax and pressed it with my hand and secured a rough impression. When he came the next time I took an impression regularly in Stent's composition, and, on examination, found the portions were fairly even on each side. When he came again, I secured an impression of one side, it being impossible to use an impression-cup for the entire jaw. Afterwards I made an impression of the other side. I took my model, separated it from the first impression, and introduced the model of the second impression; then I poured plaster into the last impression and obtained this. In a few days I had the man call again, and with this model I instructed him in working the apparatus. He was not a very intelligent man. At the end of a month's time this is what I obtained. I separated a little more and I again took an impression. I enlarged the jaw and could get the impression-cup into it, and made him a fixture such as I here present.

*Dr. Evans.*—I will exhibit to you a few specimens of celluloid. There has been a question as to the durability of this material. It is, of course, not as durable as hard rubber, nor as metal, but it affords a beauty of results superior to any of them; consequently, if properly manipulated, its durability will be from eight to fifteen years. I have tested it with a great deal of satisfaction, and have found, in an examination as to the cause of failure, that it was owing largely to the finishing of the celluloid after it was baked.

Now, if it is prepared against tin, which the microscope shows by section, it produces a hardened surface; consequently, if the work is prepared in wax first and then encased in tin models, you get a result which will be lasting for a long time. Here is a case mounted and prepared for the vulcanizer. Here is a piece after it has left the vulcanizer, in which it shows that the case is really finished, and by putting it on a brush-wheel you have it polished and ready for the mouth; it will be found very much easier to work it in wax. I prefer, of course, to put it on metal and attach the teeth to the metal by celluloid. Warping will not occur if it is thoroughly seasoned, and it, therefore, should be allowed to bake at night and to stand over till next day. I prefer a dry heat produced by steam. Here is a case which has been made several years ago, and would show warping if anything would, and here is one from the same model, the only difference is in the color and carving. There is a little piece of zylonite which shows different results.

*Dr. Elliott.*—Now, gentlemen, we will ask our guests to retire while we go on with the business of the society.

